# Pulmonary benign metastasizing leiomyoma

**DOI:** 10.11604/pamj.2021.39.68.29152

**Published:** 2021-05-25

**Authors:** Lamiae Amaadour, Nawfel Mellas

**Affiliations:** 1 Department of Medical Oncology, Hassan II University Hospital of Fez, Fez, Morocco,; 2 Faculty of Medicine, University Sidi Mohamed Ben Abdellah, Fez, Morocco

**Keywords:** Benin, uterine leiomyoma, lung, metastases

## Image in medicine

Pulmonary benign metastasizing leiomyoma (PBML) is a rare hormone dependent disease that refers to benign hysteromyoma metastasizing to the lung. Lung biopsy is indispensable for the diagnosis for PBML. Therapeutic options include surgical removing of the tumor, hormonal therapy (hysterectomy, bilateral adnexectomy, and long-term oral drugs such tamoxifen, raloxifene, and aromatase inhibitors), and inferior vena cava filter implantation. A 57-years-old postmenopausal woman underwent total hysterectomy for uterine leiomyoma. Eight years later, she presented with a chronic cough and dyspnea. A thoracic computed tomography scan showed a solitary proximal nodule of 4 cm on the right lung (A). Histological examination of biopsies revealed a benign leiomyoma of the lung. As the surgery would be mutilating, a regular follow up was recommended but she lost sight of. The patient was lost of view for one year before she appears with increased cough, dyspnea and chest pain. A new computerized tomography (CT) scan showed a significant increase in size of the initial tumor of about 4 cm with compression of the right bronchus (B). Subsequently, biopsies have been performed as a leiomyosarcomatous transformation has been suspected. Histopathological analysis disclosed the diagnosis of a benign metastasizing leiomyoma of the lung. The patient was given the aromatase inhibitor (Letrozole 2.5 mg). This treatment yielded a disease progression after six months. Second line tamoxifen was then started. The disease is still stable for 19 months now and the patient will continue to be followed.

**Figure 1:**
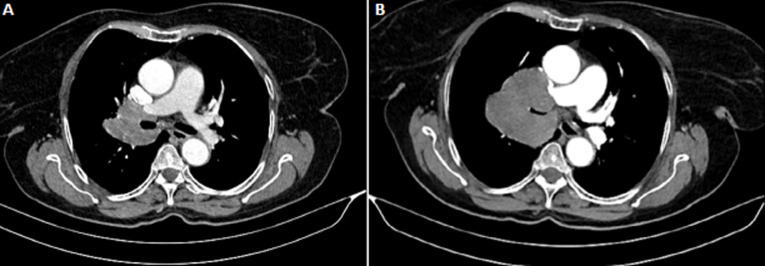
A) chest computed tomography (CT) shows a right upper lobe tissue process; B) chest computerized tomography (CT) done a year later showing an increase in the volume of the mass with compression of the bronchovascular structures

